# Tirzepatide and Glucagon-like Peptide-1/Glucose-dependent Insulinotropic Polypeptide Therapy: Quality of Life, Filipino Diet Culture, and Safe Access

**DOI:** 10.31662/jmaj.2026-0320

**Published:** 2026-07-31

**Authors:** Aaron A. Funa, Renz Alvin E. Gabay

**Affiliations:** 1 College of Health and Science, Sorsogon State University, Sorsogon City, Philippines; 2 College of Teacher Education, Sorsogon State University, Sorsogon City, Philippines

**Keywords:** Filipino diet culture, GLP-1/GIP therapy, pharmacovigilance, quality of life, safe access, tirzepatide, type 2 diabetes

## Dear Editor,

We read with interest the study by Fukaishi and Ishibashi ^(1)^ on switching from oral semaglutide to once-weekly tirzepatide in patients with type 2 diabetes. The study is valuable because diabetes therapy–related quality of life improved after switching, although glycated hemoglobin level and body weight did not significantly change at 3 months ^(1)^. This finding suggests that glucagon-like peptide-1/glucose-dependent insulinotropic polypeptide (GLP-1/GIP) therapy should be evaluated not only through metabolic outcomes but also through treatment burden, patient expectations, and daily routines.

This point is relevant to Filipino patients, whose diabetes self-management is often embedded in family meals and social eating. Filipino adult dietary patterns and nutrient intakes have been associated with cardiometabolic noncommunicable disease risk factors ^(2)^. Thus, counseling should not be limited to general advice such as “reduce carbohydrates.” It should include culturally understandable examples: smaller rice portions, healthier *ulam* or main dishes eaten with rice, fewer sugar-sweetened beverages, and planning for *handaan* or fiestas, meaning communal celebration meals, without stigmatizing patients.

The study also involved patients who switched because of insufficient weight reduction ^(1)^. This should be interpreted carefully because the weight-related effects of tirzepatide may influence patient expectations. In the Philippines, interest in “fitness” may include health, confidence, photographs, reunions, work presentation, or social media visibility. A Philippine adolescent study found that social media affinity was associated with eating attitudes and body dissatisfaction ^(3)^. Although this should not be generalized to all adults with diabetes, it supports asking whether treatment interest is motivated by metabolic goals, appearance concerns, or both.

Safe access is another important extension of the study. The benefits reported by Fukaishi and Ishibashi ^(1)^ occurred in a clinical setting, where switching and follow-up were supervised. In contrast, patients who cannot afford or access supervised therapy may turn to informal or unverified online sources. No-prescription online semaglutide purchases have been associated with unlicensed sellers, product-quality concerns, and dosing concerns ^(4)^. In the Philippines, the Food and Drug Administration has warned that online medicine selling is not generally permitted except through approved services of licensed pharmacies ^(5)^.

Future tirzepatide studies should therefore include quality of life, patient motivations, affordability, sourcing pathways, adverse events, and discontinuation. The appropriate response to unverified access should not be patient blame, but accessible supervised care, product-quality testing, transparent regulatory review, and independent pharmacovigilance. Such evidence-generating systems can help determine whether the local benefits of GLP-1/GIP therapy outweigh risks while protecting patients from unsafe self-directed use.

## Article Information

### Acknowledgments

The authors gratefully acknowledge the administration of Sorsogon State University for its institutional support. Artificial intelligence assistance was used for literature checking, drafting, and language refinement. All cited claims and references were checked against verifiable sources. The authors remain responsible for the final content.

### Author Contributions

Aaron A. Funa conceptualized the manuscript, conducted the literature search, drafted the original manuscript, and revised it for intellectual content. Renz Alvin E. Gabay contributed to literature checking, critical review, and editing. Both authors approved the submitted version and agree to be accountable for all aspects of the work.

### Conflicts of Interest

None

### Informed Consent

Not applicable.

### AI-use and Accuracy Disclosure

Artificial intelligence assistance was used for literature checking, drafting, and language refinement. All cited claims and references were checked against verifiable sources. The authors remain responsible for the final content.
